# Large-scale cross-species chemogenomic platform proposes a new drug discovery strategy of veterinary drug from herbal medicines

**DOI:** 10.1371/journal.pone.0184880

**Published:** 2017-09-15

**Authors:** Chao Huang, Yang Yang, Xuetong Chen, Chao Wang, Yan Li, Chunli Zheng, Yonghua Wang

**Affiliations:** 1 Bioinformatics Center, College of Life Sciences, Northwest A&F University, Yangling, Shaanxi, China; 2 School of Pharmacy, Shihezi University, Shihezi, China; 3 College of Economics & Management of Dalian Ocean University, Dalian, China; 4 Department of Materials Science and Chemical Engineering, Dalian University of Technology, Dalian, Liaoning, China; 5 Key Laboratory of Resource Biology and Biotechnology in Western China, Ministry of Education, School of Life Sciences, Northwest University, Xi’an, China; National Chiao Tung University College of Biological Science and Technology, TAIWAN

## Abstract

Veterinary Herbal Medicine (VHM) is a comprehensive, current, and informative discipline on the utilization of herbs in veterinary practice. Driven by chemistry but progressively directed by pharmacology and the clinical sciences, drug research has contributed more to address the needs for innovative veterinary medicine for curing animal diseases. However, research into veterinary medicine of vegetal origin in the pharmaceutical industry has reduced, owing to questions such as the short of compatibility of traditional natural-product extract libraries with high-throughput screening. Here, we present a cross-species chemogenomic screening platform to dissect the genetic basis of multifactorial diseases and to determine the most suitable points of attack for future veterinary medicines, thereby increasing the number of treatment options. First, based on critically examined pharmacology and text mining, we build a cross-species drug-likeness evaluation approach to screen the lead compounds in veterinary medicines. Second, a specific cross-species target prediction model is developed to infer drug-target connections, with the purpose of understanding how drugs work on the specific targets. Third, we focus on exploring the multiple targets interference effects of veterinary medicines by heterogeneous network convergence and modularization analysis. Finally, we manually integrate a disease pathway to test whether the cross-species chemogenomic platform could uncover the active mechanism of veterinary medicine, which is exemplified by a specific network module. We believe the proposed cross-species chemogenomic platform allows for the systematization of current and traditional knowledge of veterinary medicine and, importantly, for the application of this emerging body of knowledge to the development of new drugs for animal diseases.

## Introduction

Drug discovery aims at finding molecules that will target a specific pathway or pathogen with minimal side effects [1]. However, productivity, in terms of new drug approvals, has presumably been falling for almost a decade and the safety of a considerable number of highly effective drugs has recently been introduced into doubt [2]. For example, about 2.3 million adverse event reports were collected against ∼6000 marketed drugs between 1969 and 2002 [3]. Therefore, the pharmaceutical industry is presently beleaguered by detailed scrutiny from the financial sector, managers and the wider population [2]. To achieve the potential for rescuing the pharmaceutical industry, shifting the focus of drug discovery from chemosynthesis to cross-species sources, typically natural products from medicinal plants, is essential for discovering effective therapeutic agents that revolutionized treatment of serious animal diseases.

Medicinal plants are a vital source of phytochemicals that supply traditional medicinal treatment of various diseases [4]. At present, the interest in medicinal plants has increased significantly in animal therapy, which is named as VHM [5]. As described by Viegi et al. [6], cattle, horses, sheep, goats and pigs account for about 70% of the animals cured with herbal remedies, followed by poultry (9.1%), dogs (5.3%) and rabbits (4.3%). This is not only because of a general trend towards the utilization of natural products for therapeutic diseases but also attributable to the availability of extensive evidence regarding the efficacy of herbal remedies [7]. A case in point is ‘Zoopharmacognosy’, which refers to animals self-medicate by searching for herbs best capable of treating their disease [8, 9]. Although the clinical efficiency and safety of herbs are unquestioned for animal disease, identification of the new structural leads remains a matter of dispute. This raises questions about whether these most successful source of drugs (natural products) has any place in modern drug discovery [10, 11].

With the above background, it is worth considering how new drugs have been discovered. In general, three different type approaches have been, and continue to be utilized. These are: traditional, empirical and experimental. The traditional approach takes advantage of material that has been discovered by years of trial and error in dissimilar medical system. Typical examples cover drugs such as morphine, quinine and ephedrine that have been widely and long-term used, and the closest adopted compounds such as the antimalarial artemisinin. The empirical approach constructs on an interpretation of a correlative physiological process and regularly exploits a therapeutic agent from a naturally occurring lead molecule. Representative drugs include muscle relaxant tubocurarine, β-adrenoceptor antagonist propranolol, and histamine H2 receptor antagonist cimetidine [10]. The drawback of this approach is that it lacks the scientific and standard evaluation system of modern medicine. The experimental approach is based on the development of molecular biological techniques and the advances in genomics. The majority of drug discovery is currently on the basis of the experimental approach, which is unfortunatly time-consuming and laborious [12]. Thus, a new approach, such as computer strategies, will be needed to remedy this situation.

More recently, the advent of–omics technologies that rapidly measure the entirety of the complement of various organisms, for example, genes (genomics) or metabolites (metabonomics)—and to integrate these diverse data into a complete picture—has given rise to a new way of looking at the herbal remedies in the form of chemogenomic profile [13]. Chemogenomics is an incipient discipline that integrates the latest instruments of genomics and chemistry and applies them to target and drug discovery. Its strength lies in eliminating the bottleneck that presently arises in target identification by measuring the wide, conditional effects of chemical libraries on entire biological systems or by filtering huge chemical libraries rapidly and effectively against given targets. The hope is that chemogenomics will concurrently recognize and verify therapeutic targets and detect drug candidates to quickly and efficiently generate new drugs for many diseases [14].

**Fig 1:**
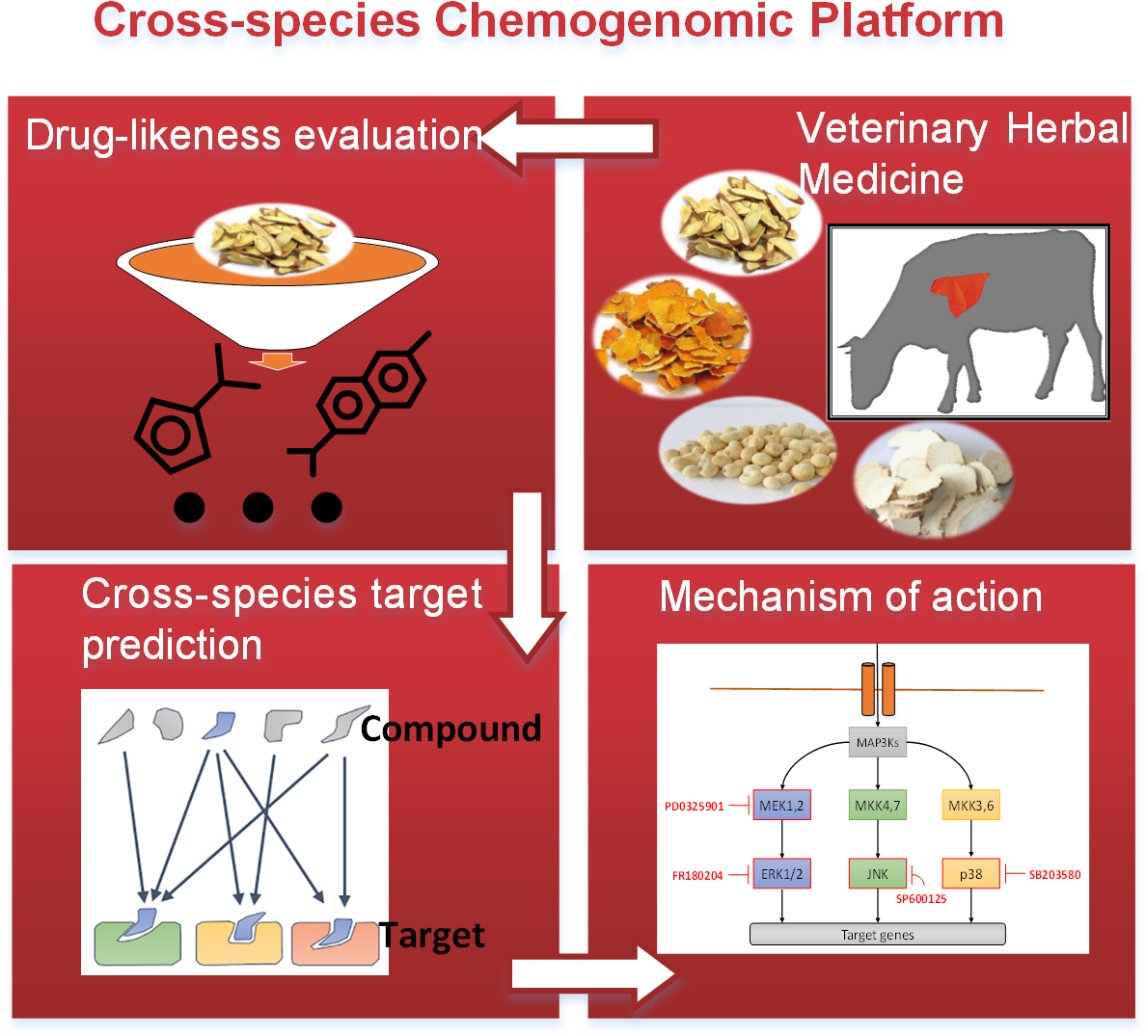
Flowchart of the cross-species chemogenomic platform.

In this study, we construct a cross-species chemogenomic screening platform to decode the drug discovery procedure and utilized it into VHM, which is exemplified by identifying lead compounds that have curative effect on Bovine pneumonia of erchen decoction (Fig 1). This herbal remedy is a China proved prescription for the treatment of pneumonia, which is composed of *Pinellia ternata (Thunb*.*) Breit* (*Pinellia ternate*), *Tangerine Peel*, *Poria cocos* (*Schw*.) *Wolf* (*Tuckahoe*) and *Glycyrrhiza uralensis Fisch* (*Licorice*). First, based on critically examined pharmacology knowledge, we propose a large-scale statistical analysis to evaluate the efficiency of ingredients in herbal remedy, which consists of drug-likeness (DL) assessment and chemical properties comparison. Second, specific informatics method is developed based on complex structure-, omics- analysis to infer drug-target connections, with purpose to understand how drugs work on the specific targets. Third, we focus on the exploration of the interactions among active ingredients, targets and disease by carrying out network-based systematic investigations, such as network convergence and modularized analysis. Finally, we choose a typical convergent module and associate it with pathway to reveal the molecular basis of the therapeutic potential. We believe the large-scale cross-species chemogenomic platform promise to improve decision making in pharmaceutical development and announce the mechanism of action.

## Materials and methods

### Data sets

All the compounds in erchen decoction are collected from the TCMSP database[15].

### DL assessment

DL is calculated by Tanimoto similarity [16] between herbal compounds and the average molecular properties of all veterinary drugs in FDA. The molecular properties refer to the 1,664 symbols which are calculated by Dragon professional version 5.4. The 1,664 descriptors are divided into 20 different types, such as constitutional, topological, 2D-autocorrelations, geometrical and so on. After removing the descriptors that are not available for all drugs, 1,533 descriptors are finally used (S1 Table).

$$T(A,B)=\frac{A\cdot B}{{\left\|A\right\|}^{2}+{\left\|B\right\|}^{2}-A\cdot B}$$

where A is the molecular descriptors of herbal compounds, B represent the average molecular properties of all veterinary drugs in FDA. In this work, ingredients with DL ≥ 0.15 are regarded as the candidate bioactive molecules, because the mean value of DL for all veterinary drugs in FDA is 0.15 (S1 Fig).

### Physicochemical features calculation

Molecular weight (MW), number of hydrogen bond acceptors (nHAcc), number of hydrogen bond donors (nHDon), octanol-water partition coefficient (MlogP) and number of rotatable bonds (RBN) these physicochemical parameters are calculated by the Dragon software [17] in this work. According to the Lipinski’s rule of five, the threshold value of them are respectively set to: 500, 10, 5, 5 and 10.

### Target identification

In an effort to predict the therapeutic target of animals, we construct a novel cross-species target prediction model (CSDT) by using Random Forest [18], which expands the predicted protein scope to all Swiss-Prot in Uniprot database [19], including 549,649 sequences involving 13,241 species such as Eukaryotes, Procaryotes, and Viruses. The building mainly includes the following four steps (S2 Fig):

1. *Benchmark Dataset*. Drug-target interactions are retrieved from the DrugBank database (http://www.drugbank.ca/, accessed on October 1, 2015). To eliminate noise of this data set, we further match them to STIICH [20], SuperTarget [21] and KEGG [22] database. In total, a 12,907 drug-target Interactions including 5,689 drugs and 3,650 targets is applied in this work as the benchmark dataset (S2 Table);
2. *Descriptor calculation*. To characterize the drugs and targets with known pharmacological interactions, drug structures and protein sequences are converted into numerical descriptors by employing DRAGON program (http://www.talete.mi.it/index.htm) and ProteinEncoding (http://jing.cz3.nus.edu.sg/cgi-bin/prof/prof.cgi/), respectively. As a result, each drug is represented as 900 physicochemical descriptors. For a certain protein, it is characterize by 1,545 dimensions structural and physicochemical features (S3 Table);
3. *Construction of training and test sets*. The positive set is constructed by the known drug-target interactions that extracted from the DrugBank database [23]. The negative set is assembled by a random generation of the same number of relations that do not overlap with those positive interactions, which is repeated 1,000 times to overcome the choice bias of the negative set. For each time, the dataset is then randomly split into two subsets, i.e., training set (19,360 = 9,680 positive interactions+9680 negative interactions, 3/4 of total sets) used to construct the model and an independent test set (6,454 = 3,227positive interactions+3,227negative interactions, 1/4 of total sets) to validate the accuracy of the model. Finally, these data are applied for random forests (RF) (http://www.stat.berkeley.edu/users/breiman/) modeling process. Default settings are used for the parameters: 500 for the number of trees and the square root of the total number of variables for the number of randomly selected variables, respectively.
4. *Model performance*. With the purpose of deriving a reliable *in silico* model, both internal and external validation methods are applied. For the internal validation, the target prediction model is evaluated and verified with 5-fold cross-validation. The training set is firstly randomly separated into five approximately equal-sized subsets, where four subsets are selected as the training set to build a model and the remaining samples as test set. This process is repeated five times to ensure every subset can be predicted as a validation set once. As a result, the derived model performs well in predicting the drug targets with the accuracy of 77.04±0.80%, the sensitivity of 75.3±1.10%, the specificity of 77.48±0.98%, and the area under the receiver operating curves (AUC) of 0.86±0.01 (S3 Fig), respectively. For the external validation, the model shows the accuracy of 75.81±1.31%, the sensitivity of 74.27±1.67%, the specificity of 76.30±1.48%, and the AUC of 0.85±0.12 (S3 Fig).

### Drug direct targeting

We also apply the ensemble similarity (WES) algorithm [24] to identify direct targets of ingredients in erchen decoction. WES quantitatively evaluates whether a molecule will direct bind to a target based on the weighted structural and physicochemical features it shares with known ligands of the target. The WES model performs well in predicting the binding (sensitivity 85%, SEN) and the nonbinding (specificity 71%, SPE) patterns, with the accuracy of 78%, the precision (PRE 74%) and the area under the receiver operating curves (AUC) of 0.85, respectively.

### GO and KEGG pathway enrichment

We utilize the DAVID [25] to decipher the biological interpretation of the predicted targets of erchen decoction.

### Network construction

Target-target (T-T) interaction are built by searching the STRING database. Specifically, in the STRING database, the target-target interactions are respectively given a confidence score: high confidence (0.7), medium confidence (0.4) and low confidence (0.15). To ensure the accuracy of the obtained target-target interactions, we search the STRING database with the confidence (score) greater than or equal to 0.7. The compound-target network and target-target network are displayed by Cytoscape 3.3 [26]. Cytoscape is a popular bioinformatics package for biological network visualization and data integration.

## Results and discussion

### Identification lead compounds through cross-species drug-likeness evaluation

Multicomponent quantitative analysis is one of the mainstream quality control methods of herbal medicines, since the ingredients of herbal medicines materials are heterogeneous [27]. In this work, 493 chemical components of erchen decoction are extracted from our database TCMSP (http://lsp.nwu.edu.cn/tcmsp.php). TCMSP is a unique systems pharmacology platform of Chinese herbal medicines that captures the relationships between drugs, targets and diseases [15].

To efficiently remove compounds chemically unsuitable for veterinary drug discovery, we construct a cross-species drug-likeness evaluation method based on Tanimoto coefficient [16] (see materials and methods). Here, DL is a complicated balance of diverse molecular properties and structure features which govern whether a particular molecule in erchen decoction is analogous to the known veterinary drugs in FDA (http://www.fda.gov/). And, the filtering criteria is defined as DL ≥ 0.15, because the average value of DL for all 333 veterinary drugs in FDA is 0.15. In total, among the 493 compounds, 126 representative compounds with favorable DL value are singled out and displayed in Table 1. Note that 48% (61/126) of the active agents have been reported by literatures S4 Table. For example, baicalin in *Pinellia ternate* protects mice from Staphylococcus aureus pneumonia via inhibition of the cytolytic activity of α-hemolysin [28]. Cavidine possesses anti-inflammatory activity and has been used to treat various inflammatory diseases [29]. These results indicate that the DL prediction approach is not only easy to discover known active ingredient, but also available to predict potential active ingredients.

**Table 1:** Candidate active compounds.

No.	Herb	Compound Name	DL	MW	MLOGP	nHDon	nHAcc	RBN
M017	Tuckahoe	Cerevisterol	0.18	430.74	5.15	3	3	4
M020	Tangerine Peel	beta-Citraurin	0.16	432.7	7.1	1	2	9
M024	Tuckahoe	3-oxo-6, 16α-dihydroxylanosta-7, 9, 26-trien-23-oicacid	0.27	502.81	5.01	3	5	6
M030	Licorice	licorice-saponin B2	0.33	809.06	3.35	8	15	7
M033	Pinellia ternate	soya-cerebroside ii_qt	0.34	552	10.32	4	5	29
M034	Pinellia ternate	soya-cerebroside I_qt	0.34	552	10.32	4	5	29
M043	Tuckahoe	poricoic acid DM	0.25	528.8	4.98	3	6	11
M058	Licorice	Licorice glycoside A	0.78	726.74	1.81	8	16	14
M068	Licorice	Gancaonin H	0.16	420.49	4.71	3	6	3
M075	Licorice	Isoglycyrol	0.17	366.39	4.36	1	6	1
M079	Licorice	Hispaglabridin B	0.18	390.51	5	1	4	1
M085	Licorice	kanzonols L	0.23	488.62	6.57	3	6	5
M090	Pinellia ternate	campesterol	0.15	400.76	7.97	1	1	5
**M114**	**Tangerine Peel**	**naringin**	**0.39**	**580.59**	**-0.47**	**8**	**14**	**6**
M118	Tuckahoe	poricoic acid D	0.23	514.77	4.73	4	6	10
M123	Tuckahoe	Ergosterol	0.15	396.72	6.93	1	1	4
**M136**	**Licorice**	**Araboglycyrrhizin**	**0.39**	**779.03**	**2.72**	**7**	**14**	**6**
M140	Tuckahoe	Dehydroeburiconic acid	0.23	466.77	6.85	1	3	6
M149	Licorice	18α-hydroxyglycyrrhetic acid	0.33	486.76	4.55	3	5	1
M154	Licorice	Xambioona	0.17	388.49	4.68	0	4	1
M155	Licorice	Vicenin-2	0.35	594.57	-2.45	11	15	5
M175	Licorice	Liquiritin apioside	0.36	550.56	-0.75	7	13	7
M187	Tuckahoe	polysaccharides	0.17	504.5	-6.01	11	16	7
M188	Tuckahoe	Beta-Glucan	0.17	504.5	-6.01	11	16	7
M207	Tuckahoe	poricoic acid H	0.21	500.79	6.39	3	5	10
**M214**	**Licorice**	**licorice glycoside E**	**0.59**	**693.71**	**1.59**	**7**	**14**	**10**
M222	Licorice	licorice-saponin G2_qt	0.34	486.76	4.4	3	5	2
M223	Licorice	24-Hydroxyglycyrrhetic acid	0.34	486.76	4.4	3	5	2
M225	Licorice	Docosyl caffeate	0.29	488.83	11.16	2	4	24
M238	Pinellia ternate	Stigmasterol	0.17	412.77	7.64	1	1	5
M243	Licorice	Artonin E	0.18	436.49	4.67	4	7	3
M249	Licorice	licorice-saponin F3_qt	0.34	454.76	5.93	1	3	0
**M267**	**Pinellia ternate**	**soya-cerebroside I**	**0.62**	**714.16**	**8.57**	**7**	**10**	**32**
**M268**	**Pinellia ternate**	**soya-cerebroside ii**	**0.61**	**714.16**	**8.57**	**7**	**10**	**32**
**M289**	**Licorice**	**rutin**	**0.46**	**610.57**	**-1.45**	**10**	**16**	**6**
M290	Pinellia ternate	Baicalin	0.16	446.39	0.64	6	11	4
M297	Licorice	Kanzonol Z	0.15	406.51	4.93	2	5	3
M299	Licorice	licorice-saponin K2	0.33	823.04	2.01	9	16	8
M310	Licorice	2’,7-Dihydroxy-4’-methoxyisoflavan-7-O-β-d-glucopyranoside	0.15	434.48	0.59	5	9	5
M317	Licorice	glycyrrhetol	0.28	456.78	5.28	2	3	1
M328	Licorice	Astragalin	0.16	448.41	-0.32	7	11	4
M331	Licorice	Kanzonol H	0.17	424.58	6.1	2	5	4
M334	Pinellia ternate	Cavidine	0.16	353.45	3.72	0	5	2
M340	Licorice	11-deoxyglycyrrhetic acid	0.29	456.78	6.42	2	3	1
M343	Tangerine Peel	Limonin	0.36	470.56	1.42	0	8	1
M352	Licorice	uralsaponin B	0.33	823.04	2.42	8	16	7
M355	Licorice	24-Hydroxy-11-deoxyglycyrrhetic acid	0.29	458.75	5.13	3	4	1
M366	Tuckahoe	Polyporenic acid C	0.25	482.77	5.68	2	4	6
M367	Tuckahoe	(16α)-16-Hydroxy-24-methylene-3-oxolanosta-7,9(11)-dien-21-oic acid	0.25	482.77	5.68	2	4	6
M369	Tangerine Peel	Tangeraxanthin	0.26	484.78	7.65	1	2	10
M376	Licorice	4H-1-Benzopyran-4-one, 2-(4-(beta-D-glucopyranosyloxy)phenyl)-2,3-dihydro-5,7-dihydroxy-, (2S)-	0.17	434.43	0.39	6	10	4
M381	Tuckahoe	Poricoic acid A	0.21	498.77	5.94	3	5	10
M392	Pinellia ternate	beta-Sitosterol	0.17	414.79	8.08	1	1	6
M394	Tangerine Peel	beta-Sitosterol	0.17	414.79	8.08	1	1	6
M395	Tuckahoe	Daucosterol_qt	0.17	414.79	8.08	1	1	6
M399	Tuckahoe	3-epidehydrotumulosic acid	0.25	484.79	5.72	3	4	6
M400	Tuckahoe	dehydrotumulosic acid	0.25	484.79	5.72	3	4	6
M424	Licorice	glycyrrhizin	0.33	823.04	2.42	8	16	7
M425	Licorice	glycyrrhizic acid	0.33	823.04	2.42	8	16	7
M426	Licorice	licorice-saponin H2	0.33	823.04	2.42	8	16	7
M451	Licorice	Ononin	0.16	430.44	0.68	4	9	5
M454	Tuckahoe	Oleanolic acid	0.28	456.78	6.42	2	3	1
**M455**	**Licorice**	**3,22-Dihydroxy-11-oxo-delta(12)-oleanene-27-alpha-methoxycarbonyl-29-oic acid**	**0.42**	**512.75**	**4.37**	**1**	**6**	**2**
M462	Licorice	licorice-saponin H2_qt	0.31	470.76	5.49	2	4	1
M463	Licorice	18β-glycyrrhetic acid	0.31	470.76	5.49	2	4	1
M464	Licorice	apioglycyrrhizin_qt	0.31	470.76	5.49	2	4	1
M465	Licorice	Araboglycyrrhizin_qt	0.31	470.76	5.49	2	4	1
M466	Licorice	glycyrrhetinic acid	0.31	470.76	5.49	2	4	1
M471	Licorice	violanthin	0.32	578.57	-1.56	10	14	4
M479	Tuckahoe	Trametenolic acid	0.21	456.78	7.03	2	3	5
M480	Licorice	isoglabrolide	0.35	468.74	5.15	1	4	0
M484	Tuckahoe	25-hydroxy-3-epidehydrotumulosic acid	0.28	514.82	4.78	4	5	6
M491	Licorice	liquoric acid	0.37	484.74	4.05	2	5	1
M493	Licorice	schaftoside	0.38	596.54	-1.5	10	16	6
M496	Tuckahoe	pachyman	0.16	500.56	-4.27	9	14	7
M497	Licorice	licuraside	0.31	550.56	-0.41	8	13	9
**M509**	**Tuckahoe**	**Daucosterol**	**0.49**	**576.95**	**6.34**	**4**	**6**	**9**
**M511**	**Pinellia ternate**	**Daucosterol**	**0.49**	**576.95**	**6.34**	**4**	**6**	**9**
M523	Tuckahoe	Poricoic acid B	0.19	484.74	5.64	3	5	9
M543	Licorice	glyasperin E	0.16	444.51	6.41	2	6	6
M546	Tuckahoe	Dehydroeburicoic acid	0.23	468.79	6.89	2	3	6
M547	Pinellia ternate	Cycloartenol	0.21	426.8	7.55	1	1	4
**M561**	**Licorice**	**3β-formylglabrolide**	**0.41**	**496.75**	**5.33**	**0**	**5**	**2**
M563	Licorice	Isoschaftoside	0.3	564.54	-1.94	10	14	4
M565	Licorice	Hirsutrin	0.18	464.41	-0.59	8	12	4
M597	Licorice	(-)-Medicocarpin	0.23	432.46	0.75	4	9	4
**M598**	**Pinellia ternate**	**TRIPALMITIN**	**0.84**	**807.49**	**19.52**	**0**	**6**	**50**
M609	Tuckahoe	(3β)-3-Hydroxylanosta-7,9(11),24-trien-21-oic acid	0.21	454.76	6.58	2	3	5
M611	Tuckahoe	poricoic acid C	0.19	482.77	7.11	2	4	10
M612	Licorice	Mairin	0.27	456.78	6.52	2	3	2
M625	Tuckahoe	Ergosta-7,22-dien-3-ol	0.15	398.74	7.18	1	1	4
**M633**	**Tangerine Peel**	**hesperidin**	**0.48**	**610.62**	**-0.48**	**8**	**15**	**7**
**M646**	**Licorice**	**apioglycyrrhizin**	**0.39**	**779.03**	**2.54**	**7**	**14**	**7**
**M663**	**Licorice**	**Nicotiflorin**	**0.43**	**594.57**	**-1.18**	**9**	**15**	**6**
M668	Pinellia ternate	Stigmast-4-en-3-one	0.17	412.77	8.18	0	1	6
M669	Tuckahoe	dehydropachymic acid	0.32	526.83	6.1	2	5	8
M685	Licorice	licorice-saponin J2	0.33	825.06	2.26	9	16	8
M690	Licorice	glabrolide	0.36	468.74	5	1	4	0
**M693**	**Pinellia ternate**	**1,2,3,4,6-Pentagalloyl glucose**	**0.57**	**1092.83**	**4.59**	**17**	**30**	**19**
M696	Licorice	Kanzonol F	0.25	420.54	5.3	1	5	3
M700	Licorice	licorice-saponin C2_qt	0.29	454.76	6.17	2	3	1
**M704**	**Licorice**	**glycyroside**	**0.39**	**562.57**	**-0.73**	**6**	**13**	**8**
**M708**	**Tangerine Peel**	**Neohesperidin**	**0.45**	**610.62**	**-0.48**	**8**	**15**	**7**
M709	Licorice	Isoviolanthin	0.32	578.57	-1.56	10	14	4
M712	Licorice	6″-O-acetylliquiritin	0.18	444.47	2.33	3	9	5
M728	Tuckahoe	Eburicoic acid	0.23	470.81	7.33	2	3	6
**M732**	**Licorice**	**Narcissoside**	**0.5**	**624.6**	**-1.2**	**9**	**16**	**7**
M738	Tuckahoe	beta-Amyrin acetate	0.31	468.84	7.68	0	2	2
M756	Tuckahoe	pachymic acid	0.32	528.85	6.54	2	5	8
M767	Tuckahoe	poricoic acid G	0.19	486.76	6.08	3	5	9
M768	Pinellia ternate	(+)-Isolariciresinol monoglucoside	0.21	522.6	0.35	7	11	7
**M770**	**Licorice**	**22β-acetylglabric acid**	**0.42**	**528.8**	**4.77**	**2**	**6**	**3**
M772	Licorice	licorice-saponin J2_qt	0.31	472.78	5.33	3	4	2
M776	Pinellia ternate	Ergosterol peroxide	0.2	428.72	6.73	1	3	4
M777	Tuckahoe	Ergosterol peroxide	0.2	428.72	6.73	1	3	4
M781	Licorice	licorice-saponin G2	0.32	839.04	1.33	9	17	8
M783	Licorice	Ursolic acid	0.28	456.78	6.47	2	3	1
M794	Licorice	1-Methoxyficifolinol	0.19	422.56	6.1	2	5	5
M803	Tuckahoe	29-hydroxypolyporenic acid C	0.27	498.77	4.59	3	5	7
M807	Licorice	licorice-saponin C2	0.33	807.04	3.1	8	15	7
M828	Tuckahoe	Tumulosic acid	0.25	486.81	6.16	3	4	6
M836	Licorice	Morusin	0.16	420.49	4.94	3	6	3
M844	Licorice	licorice-saponin K2_qt	0.31	470.76	5.08	3	4	2
M860	Tuckahoe	(3β,16α,17α)-3,16-Dihydroxylanosta-7,9(11),24-trien-21-oic acid	0.23	470.76	5.41	3	4	5
M864	Licorice	Isoononin	0.17	430.44	0.68	4	9	5
M895	Pinellia ternate	valeraldoxime	0.31	514.72	2.29	2	7	6

MW, nHAcc, nHDon, MlogP and RBN are the mainly pharmacophoric features that influence the behavior of molecule in a living organism, including bioavailability, transport properties, affinity to proteins, reactivity, toxicity, metabolic stability and many others [30]. Therefore, we further compare these chemical properties of the obtained potential active ingredients in erchen decoction with that of the 126 randomly selected molecules in the TCMSP database to further testify the validity and precision of the cross-species DL evaluation method.

**Fig 2:**
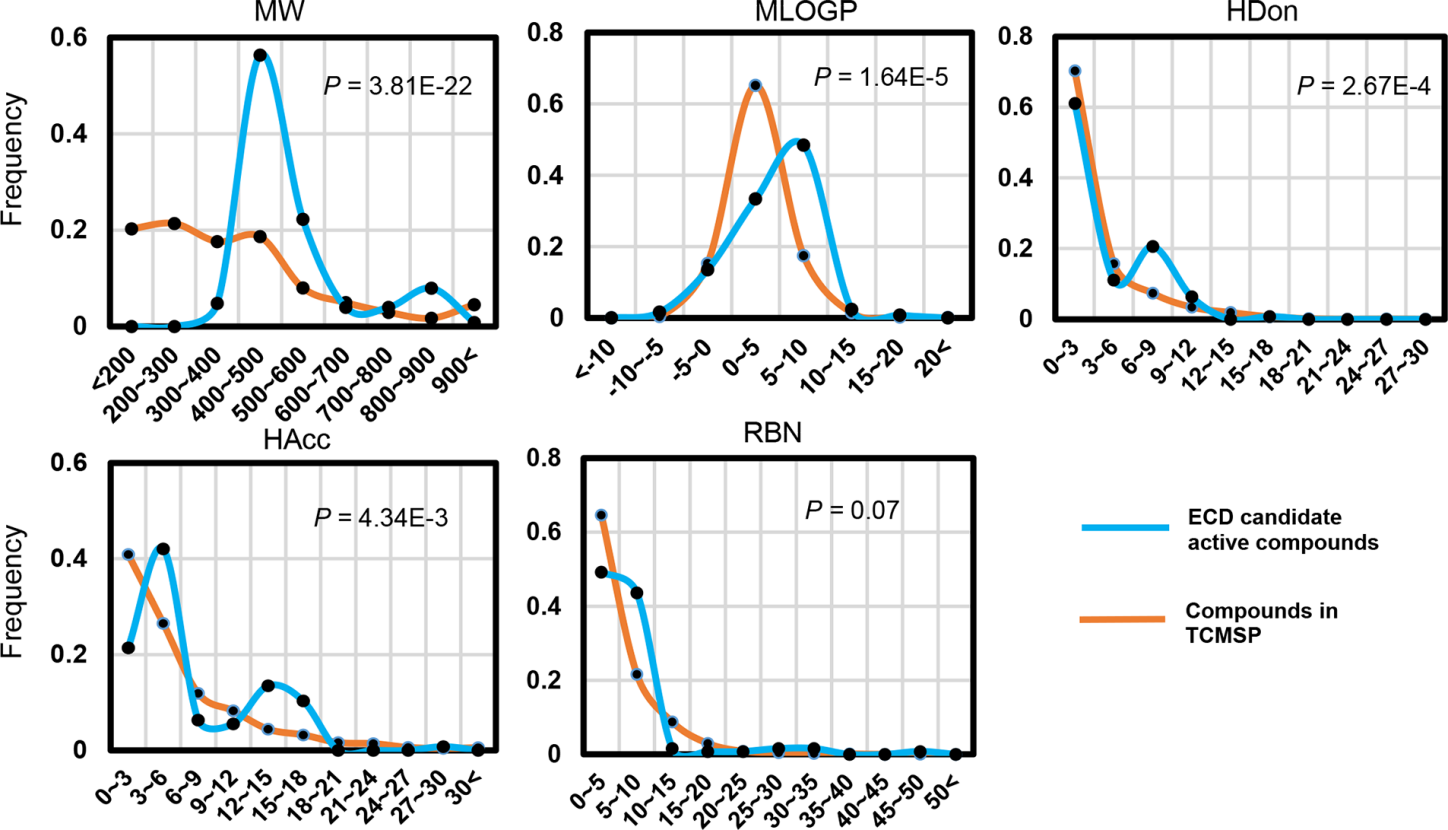
Statistics: Comparison of erchen decoction potential active compounds with equal number compounds in TCMSP database. Chemical properties of these two types of molecules are compared: distributions for molecular weight (MW), octanol-water partition coefficient (MlogP), numbers of hydrogen bond donors and acceptors (nHDon and nHAcc), and number of rotatable bonds (RBN) value are shown.

The distributions of the five pharmacological features of the above two types of ligands have different characteristics (Fig 2). Specifically speaking, a majority of the potential active compounds in erchen decoction have very low molecular weights in comparison to the ligands in TCMSP, which presumably is be caused by the fact that in proteins often very small solvent molecules are bound. Meanwhile, considerably more (40%) ingredients than TCMSP ligands fulfil the Lipinski "Rule of five" regarding the molecular weight. The same applies for RBN: 23% more active compounds in erchen decoction fulfil the Lipinski "Rule of five". A bigger percentage of active compounds in erchen decoction (90%) have less than 10 nHAcc, which is similar to that of TCMSP ligands. Meanwhile, a slightly fraction (18%) of the erchen decoction ligands have ten to twenty of them. Nevertheless, for TCMSP ligands, there are hardly no molecules meet the condition described above. Interestingly, this distribution is also applies to nHDon. Most potential active compounds in erchen (70%) have a MlogP value around 5, and the MlogP values of the TCMSP ligands accumulate around 10. Approximately 30% fraction of TCMSP ligands are "drug-like" according to the Lipinski "Rule of five", have a MlogP value less than 5. These results indicate that the cross-species DL evaluation method can reliably screen potential active ingredients.

### Prediction target proteins through the cross-species drug-target (CSDT) interaction assessment model

In the elucidation of the pharmacological activities of the filtered active ingredients in erchen decoction, knowledge of potential targets is of highest importance, which remains an ongoing focus in drug discovery efforts [31]. *In silico* prediction of such interaction is in favor of improving the efficiency of the laborious and costly experimental determination of drug-target interaction [32, 33]. However, limiting by the scope of the training datasets, both in chemical space as well as biological space, current drug-target interaction prediction models, especially ligand-based methods, seem to be all trivially adapted to make predictions for new targets of human drugs. Thus, there is still no available target prediction model for veterinary drugs.

To obtain the target proteins of the filtered active ingredients, we build a random forest [18] target prediction model, which expands the predicted protein scope to all Swiss-Prot in the Uniprot database [19], including 549,649 sequences involving 13,241 species such as Eukaryotes, Procaryotes, and Viruses (see materials and methods). The algorithm is based on extraction of conserved patterns from subdivided drug-target interaction vectors. The advantage of this model lies in that it allows us to take proteins of different species into accounts and thus predict the targets of a broad spectrum of species on a large scale. And indeed there are a similar model that we have contributed in our previous work which has been successfully applied to human target protein prediction [34]. Also, to evaluate the reliability of CSDT, we further compare the AUC of CSDT with the BATMAN-TCM [35] and HGBI method [36]. Although, the other two models outperforms CSDT, CSDT has wide adaptation range which provides help for target prediction of VHM. Thus, we can conclude that the target prediction model in this work is reliable to predict the targets that causes Bovine pneumonia. In addition, to guarantee the comprehensive of the target of active ingredients in erchen decoction, we further introduce the WES algorithm into this part [24]. WES quantitatively evaluates whether a molecule will direct bind to a target based on the weighted structural and physicochemical features it shares with known ligands of the target.

**Fig 3:**
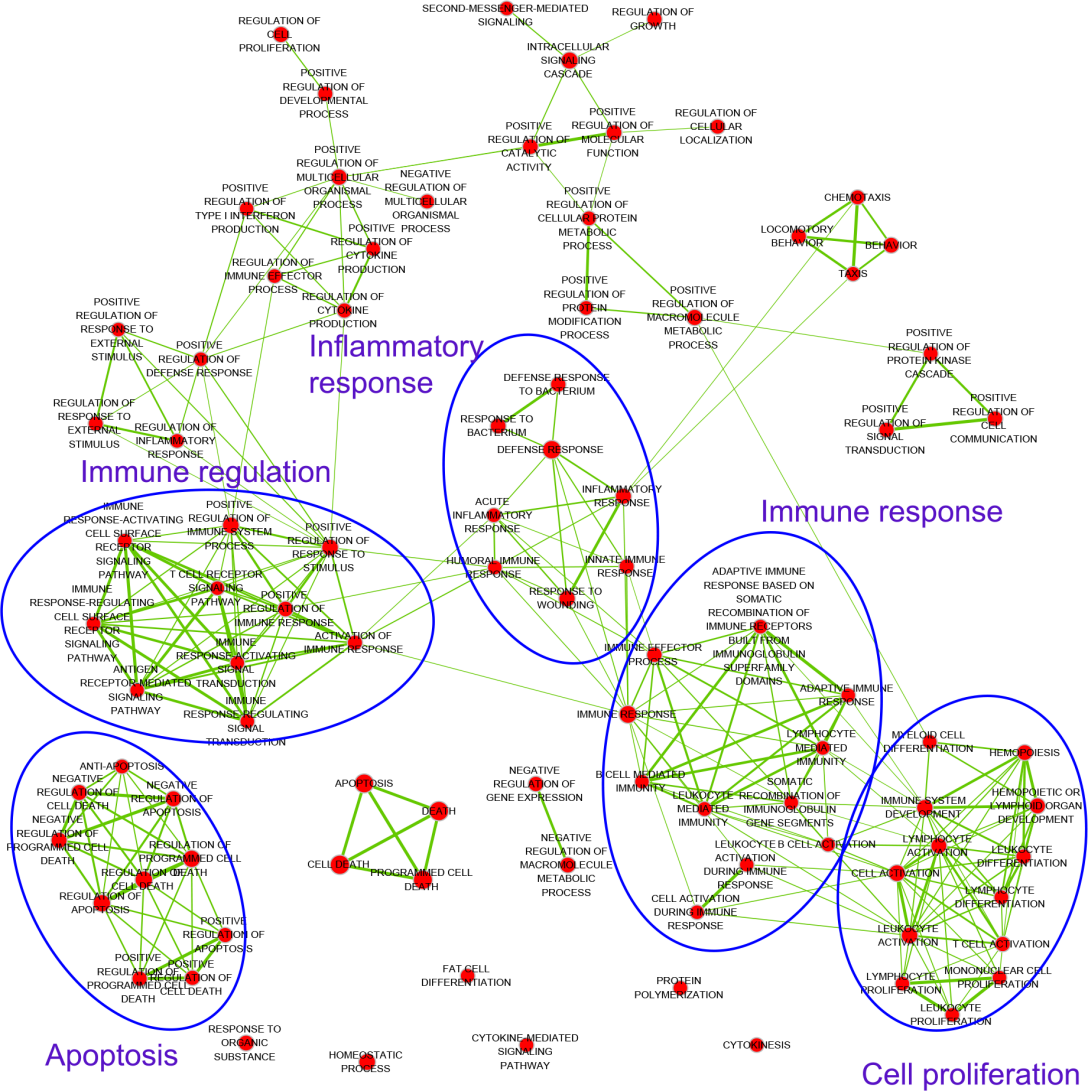
The GO biological process enrichment analysis of Bovine targets.

In total, we obtain 5,219 targets for the 126 active ingredients. Considering that the focus of our work is to obtain the targets therapeutic for Bovine pneumonia, we further restrict the species to *Bovin* (*Bos Taurus*), *STRP1* (*Streptococcus pyogenes*) and *STRPN* (*Streptococcus pneumonia*), which result in 448 targets (S5 Table). To verify whether the screened 448 targets are closely related to *Bovin* pneumonia, we respectively enrich the GO biological processes of these three types of targets by using David [25] and visualize them by enrichment Map [37] with the threshold of *P*-value ≤ 0.01. The Enrichment Map Cytoscape Plugin allows us to visualize the results of target-set enrichment as a network. GO analysis of *Bovin* targets reveal that the GO term ‘inflammatory response’ and ‘immune system process’ are significantly enriched (Fig 3 and S6 Table). Interestingly, the inflammatory response is responsible for the majority of the pulmonary damage [38]. The importance of ‘Immune system process’ in curing bacterial pneumonia is clearly demonstrated in experimental models of bovine pneumonia [39]. Erchen decoction not only targets the proteins of Bovin, but also works on the proteins of bacteria (*STRP1* and *STRPN*). For the targets of *STRP1* and *STRPN*, the main biological processes are ‘translation’, ‘tRNA metabolic process’, ‘nucleotide-excision repair’, ‘amino acid activation’, ‘tRNA aminoacylation’ and ‘ncRNA metabolic process’ (S7 Table and S8 Table). These processes are associated with cellular and metabolic processes, mainly involving in cell cycle regulation. These results suggest that erchen decoction has antibacterial activity. Taken together, the obtained targets function by directly inhibiting pathogenic bacteria proliferation through targeting their proteins essential for the bacteria life cycle, and also, indirectly suppressing bacterial infection via strengthening the immune systems of bovine.

### Recognition multiple targets interference effects by heterogeneous network convergence and modularization analysis

To identify the interrelated target set of each active ingredient in erchen decoction, we perform heterogeneous network convergence and modularization analysis in this part. Network convergence is the efficient coexistence of heterogeneous data communication within a single network. Modularization analysis is of benefit to search for functional closely related information in a biological network.

First, to discover the most potential lead compounds and decipher the action mechanism of erchen decoction, we generate two levels of networks: Compound-Target network (C-T network) and Target-Target network (T-T network). S5 Table shows a detailed view of the C-T interactions, which consists of 126 active compounds and 448 candidate targets of *Bovin*, *STRP1* and *STRPN* through 1,773 interactions. Among them, proteins such as VDR USP10 connect with more than 13 compounds, which can be labeled as hub targets. These results indicate that the distribution of the compounds is extremely inhomogeneous. Thus, intervening measures of multiple targets are of benefit to the recovery of *Bovin* pneumonia. T-T interactions are built by searching the STRING database [40] with the required confidence (score) greater than the high confidence threshold 0.7. The STRING database contains protein interactions from numerous sources, including experimental data, computational prediction methods and public text collections, which can be regarded as functional protein association networks. S9 Table provides a comprehensive view of the cross-species target space which consists of 448 nodes and 696 edges. Among these interactions, about two-thirds of the targets are regulated by at least 10 proteins, indicating the close relationship among them.

Then, we converge and modularize the aforementioned heterogeneous C-T and T-T network using Markov Cluster Algorithm (MCL) [41] implemented by clusterMaker2 for the purpose of uncovering the pharmacology correlation among the target proteins of a certain compound. ClusterMaker is a Cytoscape plugin that unifies different clustering techniques and displays into a single interface. MCL is a fast and scalable unsupervised cluster algorithm for graphs (also known as networks) based on simulation of (stochastic) flow in graphs. As a result, these interactions are mainly assigned to 11 modules, where each module contains at least 12 targets

Further, we analyze the chemical characteristics of molecules and proteins within the same modules to help us understand multiple targets interference effects of erchen. By applying the Tanimoto similarity with CDK fingerprints, we evaluate the molecular similarity among modules by comparing the molecules in different modules. The result shows that mean similarity of molecules in the same module (0.57) higher than that between modules (0.35) (one-tailed student's t-test P-value = 2.3E-213, S4A Fig). As an example, the pharmacophore model for molecules in module 1 that target TBC1D1 protein shows a good alignment to the pharmacophore and among themselves (S10 Table and S5 Fig). Similarly, to search for the common features of the proteins from the same module, we compare the similarity of protein sequences in the same module and between modules using the Smith–Waterman sequence alignment method. The similarity score is normalized by dividing it by the geometric mean of the scores obtained from the S-score of each protein against itself. We observe that the mean sequence similarity of proteins in the same module (0.031) higher than that between modules (0.021) (one-tailed student's t-test *P*-value = 1.45E-114, S4B Fig). These findings suggest that the molecules with similar structure trend to target similar targets.

**Fig 4:**
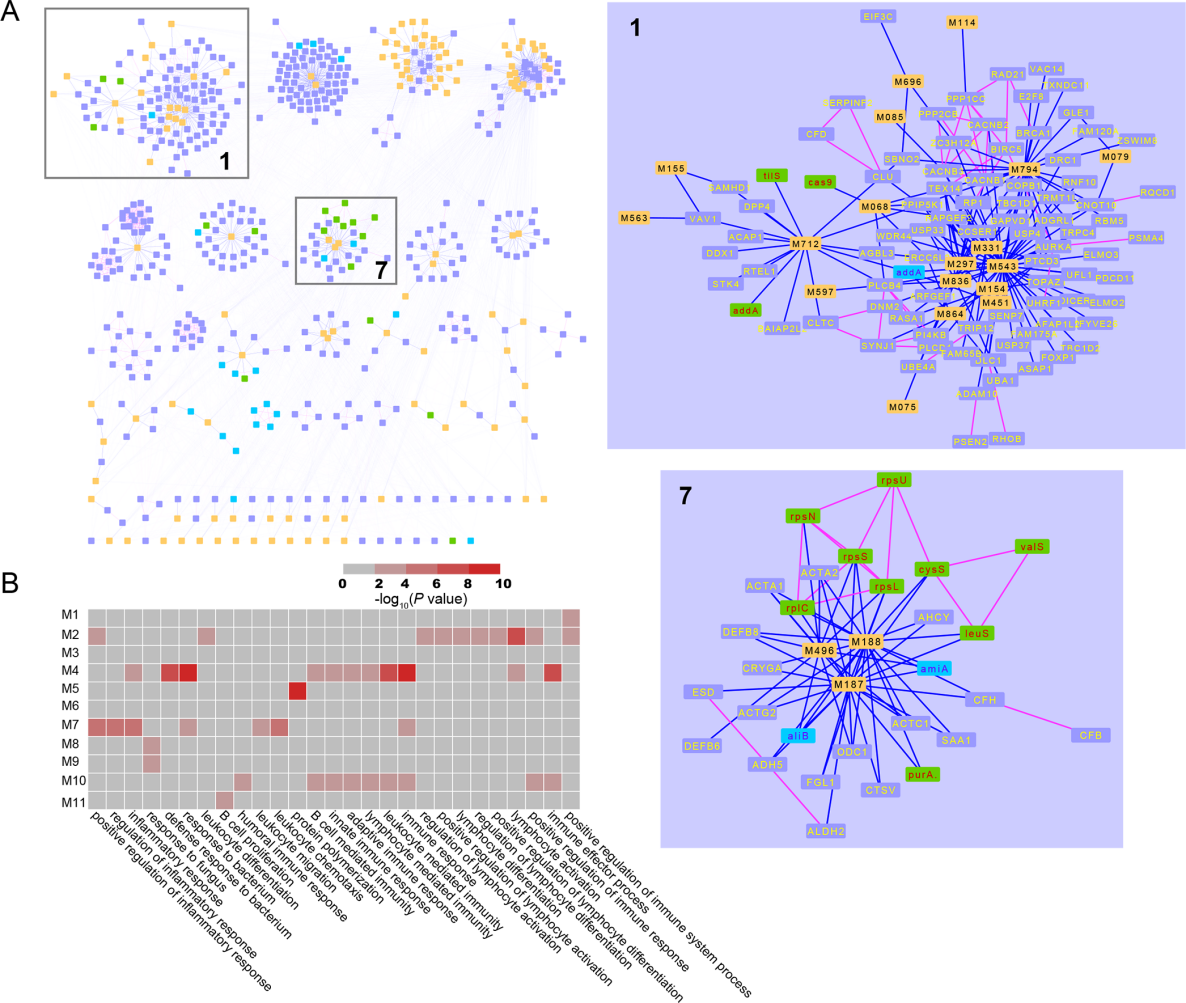
Illustration of heterogeneous network convergence and modularization analysis. (A) Global view of the modularized set of relationships among potential active compounds and their predicted protein targets. Module 1 and Module 7 are enlarged sub-modules in the global network. A compound node and a target protein node are linked if the protein is targeted by the corresponding compound. Analogous to the edge between a compound and a target, links are placed among targets if they are functional associated. Yellow node represents active compounds in erchen decoction. Green, blue and gray node respectively indicates that target of *streptococcus pyogenes*, *Streptococcus pneumonia* and Bovine.

Finally, we enrich the GO biological process and KEGG pathway of each module by David to annotate these modules. We find that these modules are associated with inflammation, immunization, and apoptosis (Fig 4B, S11 Table). We present in detail two of the converged modules (Module 1 and Module 7) (Fig 4A), selected to show the method's ability to reproduce diverse features of these compound-target interactions.

### Module 1

Module 1 reflects the 187 interactions between 18 molecules and 87 targets. The 87 targets are not only from Bovine, but also from *STRP1* and *STRPN* these two species. Among them, an overwhelming number of targets are from Bovine (95.4%, 83/87). Thus we annotate the biological process and KEGG pathway of Module 1 (S11 Table) by using these Bovine targets.

For biological process, the top 5 are respectively proteolysis involved in proteolysis involved in cellular protein catabolic process, cellular protein catabolic process, protein catabolic process, regulation of small GTPase mediated signal transduction, and cellular macromolecule catabolic process. These biological processes are all associated with protein catabolic. Interestingly, it has been reported that lung disorders where the inflammatory mediators produce direct lung damage and cause catabolism or protein degradation [42]. And therefore, the molecules in Module 1 can therapeutic for Bovine pneumonia by intervening these functionally related target proteins. For example, Vicenin-2 (M155), a flavonoid glycoside, is a potential anti-inflammatory constituent of *Licorice* [43]. Inflammatory stimuli increase SAMHD1 [44], which is a target protein of Vicenin-2. In addition, by literature research, we also observe the Bovine pneumonia associated biological function of targets in Module 1 belong to other species. For example, Cas9, a target of STRP1, can mediate bacterial immunity.

**Fig 5:**
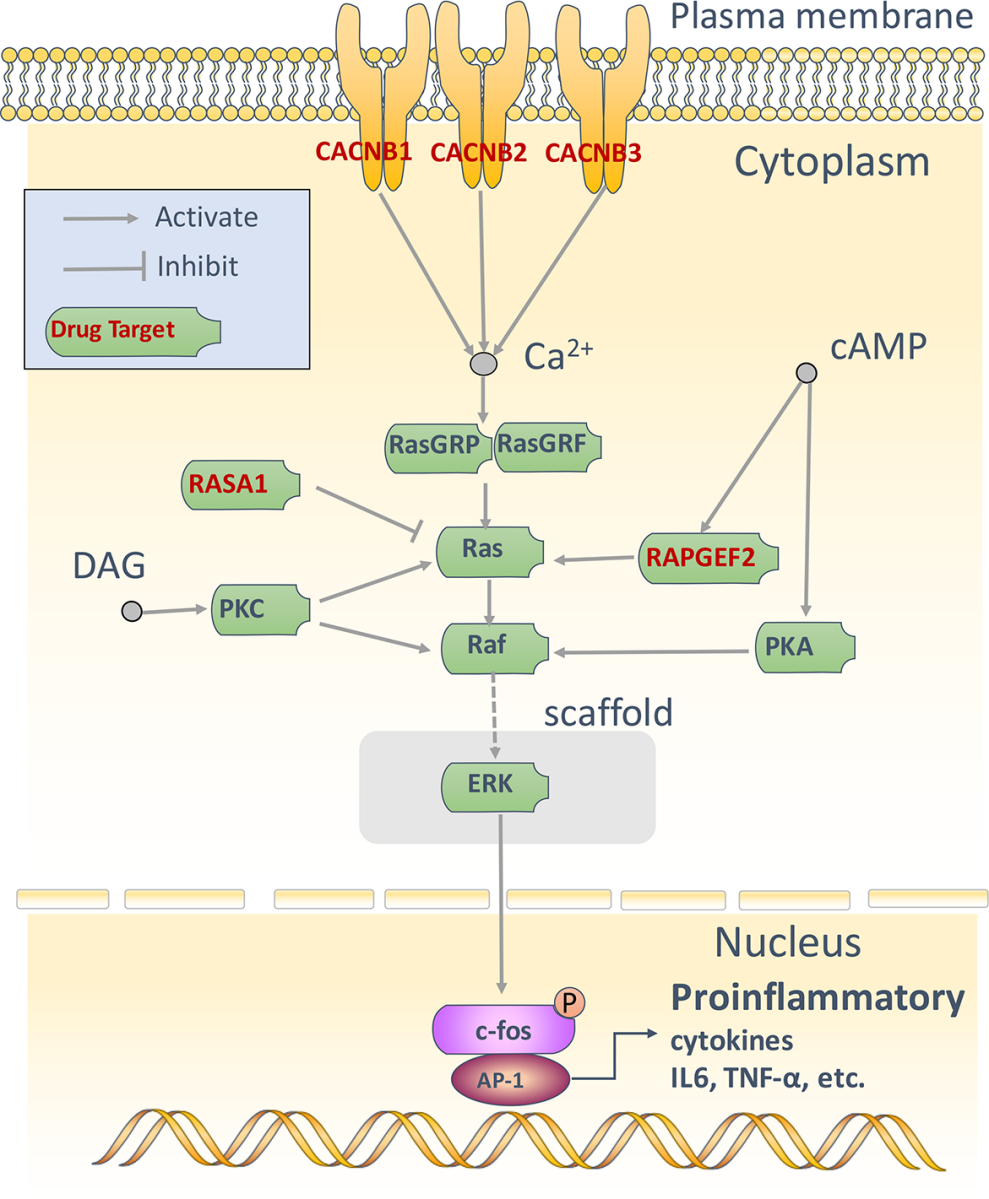
Active mechanism of erchen decoction in combating Bovine pneumonia of Module 1.

The result of KEGG pathway enrichment shows that MAPK signaling pathway, Inositol phosphate metabolism, Ubiquitin mediated proteolysis, Arrhythmogenic right ventricular cardiomyopathy (ARVC), and Cardiac muscle contraction pathway play important roles in Module 1. For example, MAPK signaling pathway (Fig 5) is a chain of proteins that plays a key role in anti-inflammatory therapy [45]. Members of Inositol phosphates metabolism pathway are a group of mono- to polyphosphorylated inositols [46]. They play crucial roles in diverse cellular functions, such as cell growth, apoptosis, cell migration, endocytosis, and cell differentiation [47]. Ubiquitin mediated proteolysis involves in the degradation of native cellular proteins [48].

### Module 7

Module 7 is an example of a converged module that covers primarily of *Bovin* genes encoding proteins (17 of 28) and, *STRP1* targets (9/28). Also, there are two *STRPN* targets in Module 7. In consideration of the number of targets in each species, we respectively enrich the biological process and KEGG pathway of *Bovin* (S11 Table) and *STRP1* (S12 Table) targets in Module 7. The results show that these two categories of targets participate in diverse metabolic pathways and cellular roles.

The *Bovin* targets in Module 7 are mainly involved in programmed cell death, cell death, death, apoptosis, and positive regulation of cellular component organization. Thus, molecules in Module 7 are intimately correlated to regulate cell apoptosis. This result is supported by a recent study that chlamydia pneumonia induces T cell apoptosis through glutathione redox imbalance and secretion of TNF-alpha [49]. In particular, Beta-Glucan (M188), which is derived from *Tuckahoe*, was predicted to target ACTA1. Beta-Glucan has been reported to inhibit the growth of bacteria, virus, and fungus [50], to stimulate macrophages as immune enhancer [51] and enhance apoptosis in human colon cancer cells SNU-C4 [52]. Interestingly, mutations in the gene ACTA1 account for cell death [53]. Also, we evaluate the 9 *STRP1* targets to test whether the proteins encoded by genes in the same module have related functions. These targets involve in many biological processes, such as translation, amino acid activation, tRNA aminoacylation for protein translation, tRNA aminoacylation, and tRNA metabolic process. In brief, these biological processes are all relevant to cell metabolic. Study shows that changes in metabolic processes play a critical role in the survival or death of cells subjected to various stresses [54]. Thus, despite the targets in the same module belong to different species, they still share homogeneous function.

The enriched KEGG pathway of *STRP1* targets are Ribosome, Aminoacyl-tRNA biosynthesis and Valine, leucine and isoleucine biosynthesis. Interfering with these pathways has effects on protein metabolism. However, deregulation of proteostasis results in protein stress and damage that may cause cell death [55]. Hence, we can conclude that KEGG pathway enrichment could also reflect the function of the module. Together, these results indicate that the strategy in this study has the ability to capture the cellular response of multiple targets interference.

## Conclusions

VHM is a holistic approach that is suited to evaluating the well-being of the whole animal, and treatments are commonly non-invasive with few side effects. Although quite new-fangled to the Western world, it is a health care system that has been used in China to treat animals for thousands of years. It is an adaptation and extension of Traditional Chinese Medicine used to heal humans. However, VHM lacks the tools necessary to identify the lead compounds which have the effect to treating animal illness. As a group with computational technology strengths, we first gravitate toward methods such as systems pharmacology [56] [24] that investigate databases or construct model for clues. The application of bioinformatics approaches enable us to elucidate the therapeutic effects of drugs at multiple scales of biological organization (the organ and organismal levels) through network analyses. And there have been a few examples of successful integration of different procedures to help determine the action mechanism of a small molecule[57] [58].

In this study, to clarify the procedure of veterinary drug discovery from herbal medicines, a cross-species chemogenomic platform was proposed. First, we build a cross-species drug-likeness evaluation approach to screen the lead compounds in veterinary medicines by critically examined pharmacology and text mining. We observe that erchen decoction can treat animal pneumonitis through multicomponent therapeutics. Furthermore, we compare the chemical properties of these molecules with equal number of randomly selected molecules. The results demonstrate that the constructed cross-species DL evaluation method is reliable to screen potentially active molecule. Second, to understand how drugs work on the specific targets, a specific cross-species target prediction model (CSDT) is developed to infer drug-target connection. In addition, by enriching the GO biological process of these targets, we find that all the biological processes of the targets are physiologically relevant. Thus, we can speculate that the active compounds in erchen decoction exert their therapeutic effect by interfering functional associated multiple targets network. To determine whether the therapeutic activity could be attributed to the selectively functional in target network, we subsequently converge the heterogeneous network and modulated analysis. Interestingly, the empirical analysis results demonstrate our scientific hypotheses. Finally, we manually characterize an integrated pathway to test whether the cross-species chemogenomic platform could uncover the active mechanism of veterinary medicine, which is exemplified by a network module.

The cross-species chemogenomic platform shows how powerful the ability to effectively and systematically integrate large sets of disparate data will be in discovering new drugs and understanding the molecular mechanisms of a small molecule in biological systems. When done in a disciplined and thoughtful manner, such data integration characterizes a modern instantiation of the scientific approach, depending on high-throughput biotechnology, data consolidation and multidisciplinary tactics to offer hints and avenues to new targets and mechanisms of small-molecule action.

## Supporting information

S1 Fig DL distribution of FDA-approved veterinary drugs. (TIF)

S2 Fig The flowchart of the CSDT model. (TIF)

S3 Fig ROC (Receiver Operating Characteristic) plot of CSDT model. (TIF)

S4 Fig (A) The frequency histogram of molecule similarity between modules (blue) and within modules (brown). Firstly, the similarity among molecules in the same module are calculated by applying the Tanimoto similarity with their CDK fingerprints. Then, using the same method, we evaluate the molecular similarity among modules by comparing the molecules in different datasets. The result shows that mean similarity of molecules in the same module (0.57) higher than that between modules (0.35) (one-tailed student's t-test P-value = 2.3E-213). (B) The frequency histogram of target sequence similarity between modules (blue) and within modules (brown). The sequence similarity between two targets are calculated based on the Smith–Waterman sequence alignment score. The similarity score is normalized by dividing it by the geometric mean of the scores obtained from the S-score of each protein against itself. The result shows that the mean sequence similarity of proteins in the same module (0.031) higher than that between modules (0.021) (one-tailed student's t-test P-value = 1.45E-114).
(TIF)

S5 Fig The alignment of compounds on the best pharmacophore model for the protein TBC1D1 in module 1. (TIF)

S1 Table Descriptors used to calculate DL. (XLS)

S2 Table The drug-target interactions in Drugbank used to build the CSDT model. (XLS)

S3 Table Descriptors used to construct the CSDT model. (XLS)

S4 Table Reference validation of candidate active compounds. (XLS)

S5 Table Compound-Target interactions. (XLS)

S6 Table GOBP and KEGG pathway enrichment results of *Bos Taurus* targets. (XLS)

S7 Table GOBP and KEGG pathway enrichment results of *Streptococcus pyogenes* targets. (XLS)

S8 Table GOBP and KEGG pathway enrichment results of *Streptococcus pneumonia* targets. (XLS)

S9 Table Target-target interactions. (XLS)

S10 Table The value of SPECIFICITY, N_HITS, FEATS and PARETO for the 20 pharmacophore model of protein TBC1D1. (DOCX)

S11 Table GOBP and KEGG pathway enrichment results of modules 1–11. (XLS)

S12 Table GOBP and KEGG pathway enrichment results of STRP1 targets in module 7. (XLS)
