# Supplementary figures and images for: Large-scale cross-species chemogenomic platform proposes a new drug discovery strategy of veterinary drug from herbal medicines
Source: PLoS One. 2017 Sep 15;12(9):e0184880. doi: 10.1371/journal.pone.0184880 (PMC5600375; doi:10.1371/journal.pone.0184880)

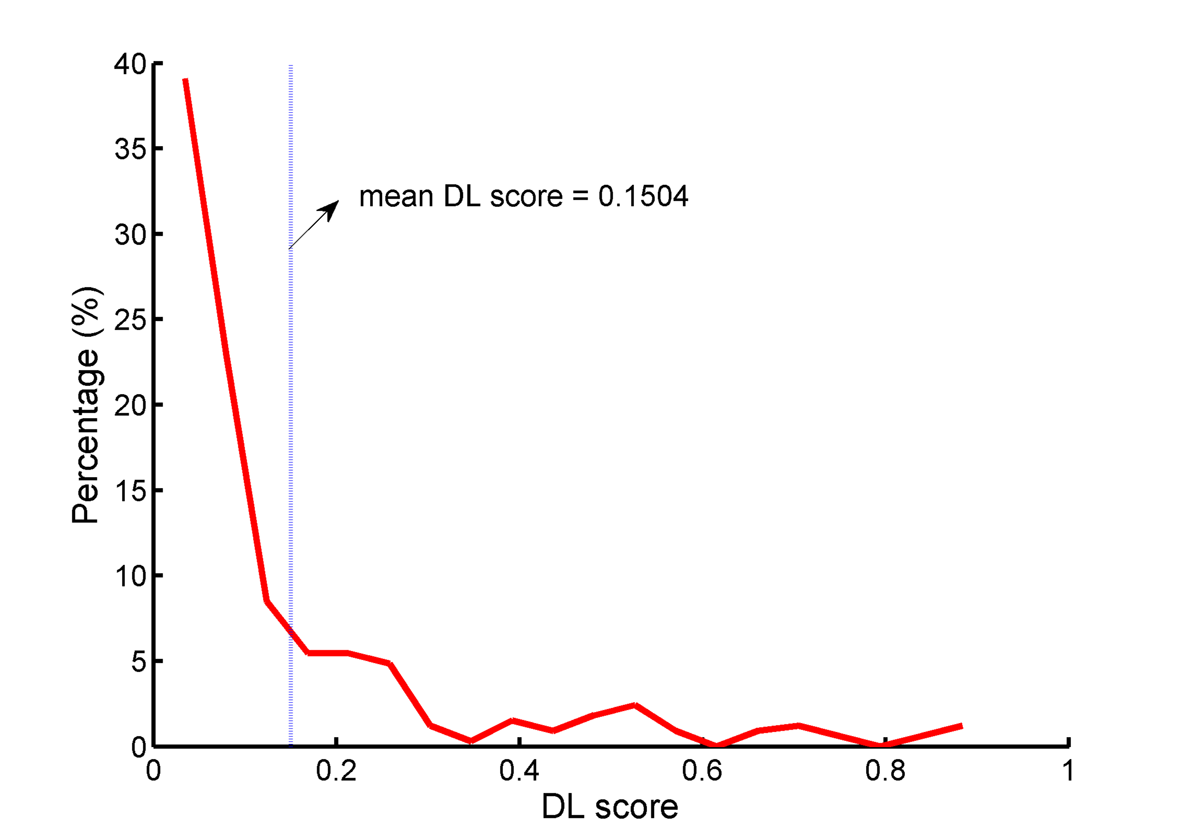

Supplement: S1 Fig — (TIF) [file pone.0184880.s001.tif]

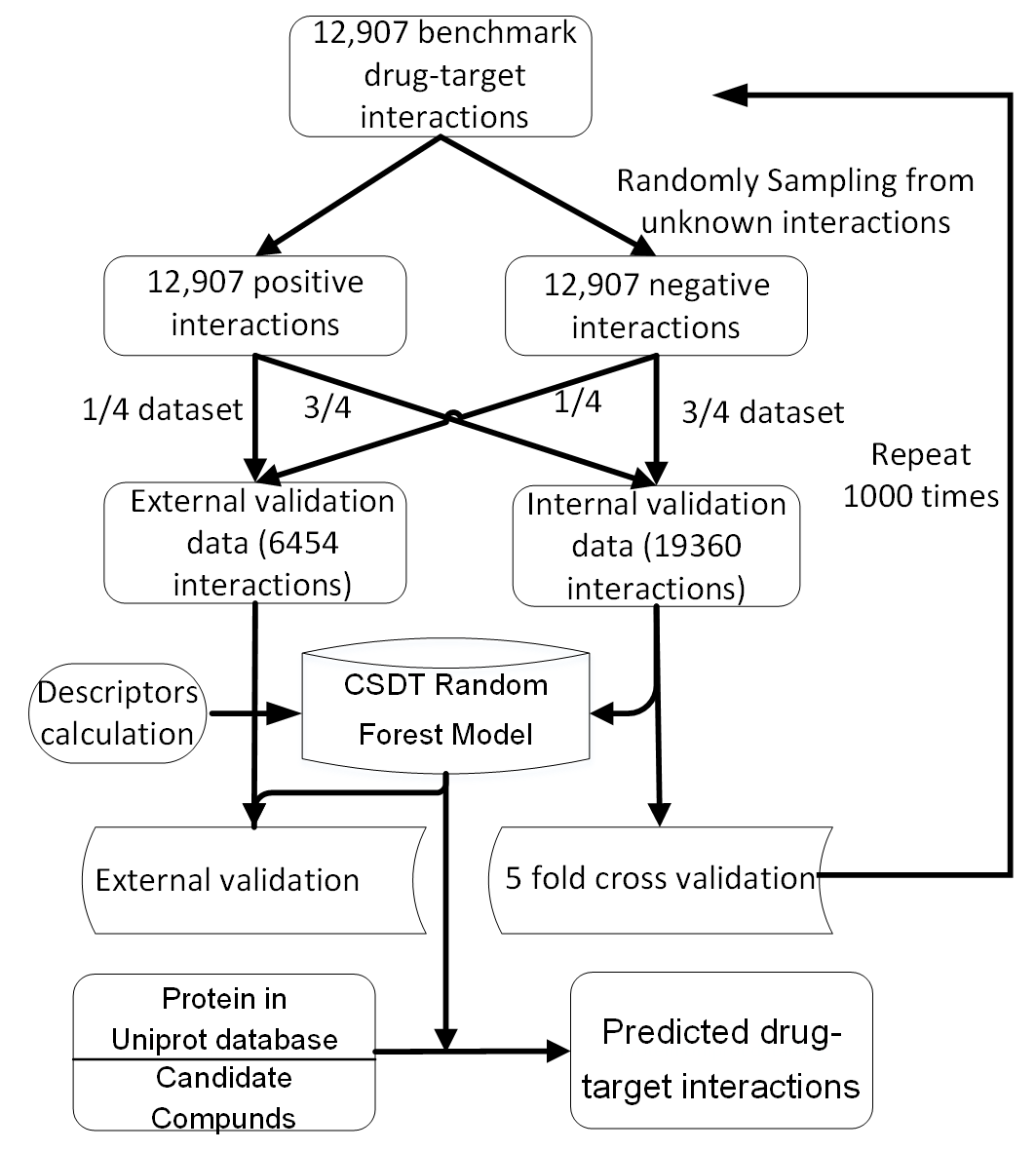

Supplement: S2 Fig — (TIF) [file pone.0184880.s002.tif]

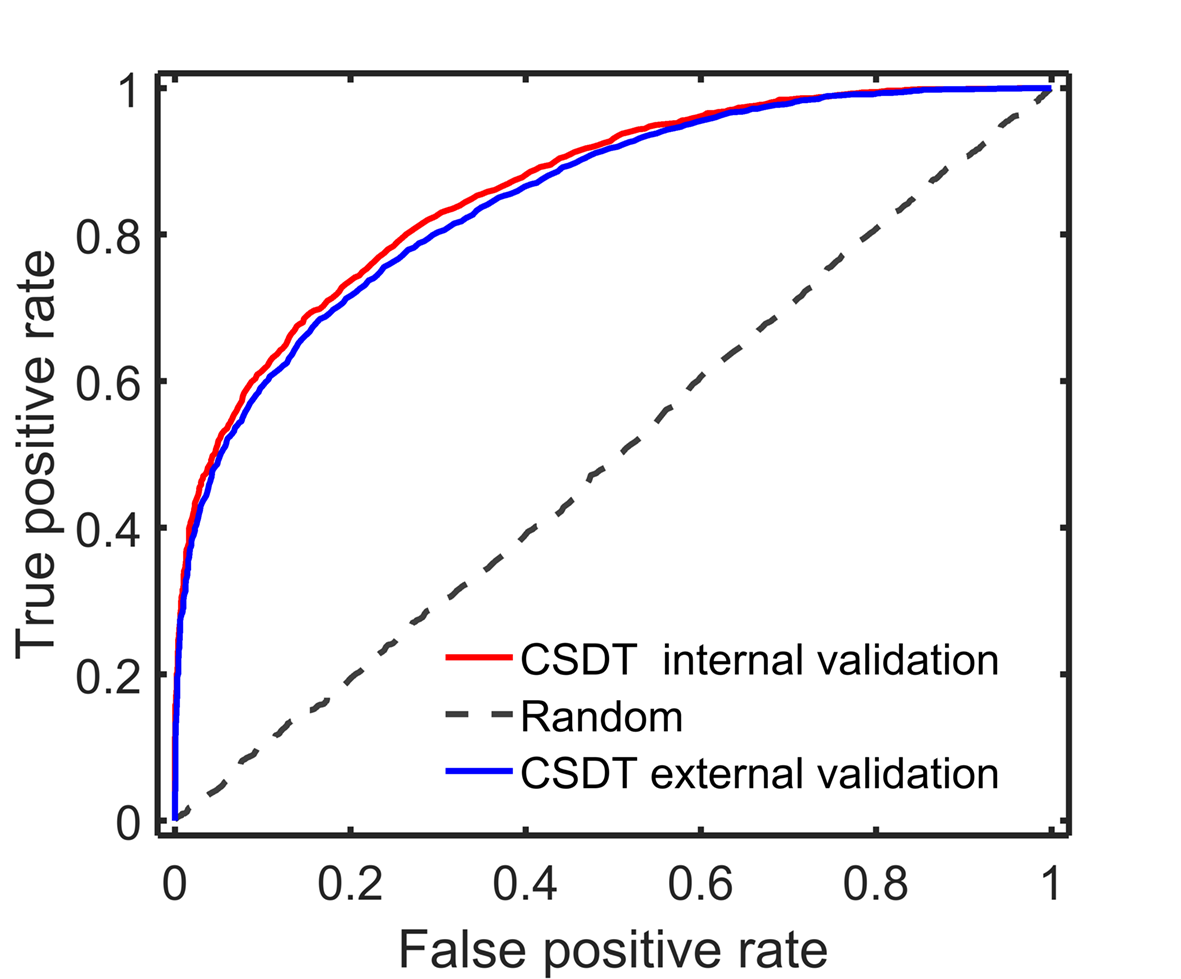

Supplement: S3 Fig — (TIF) [file pone.0184880.s003.tif]

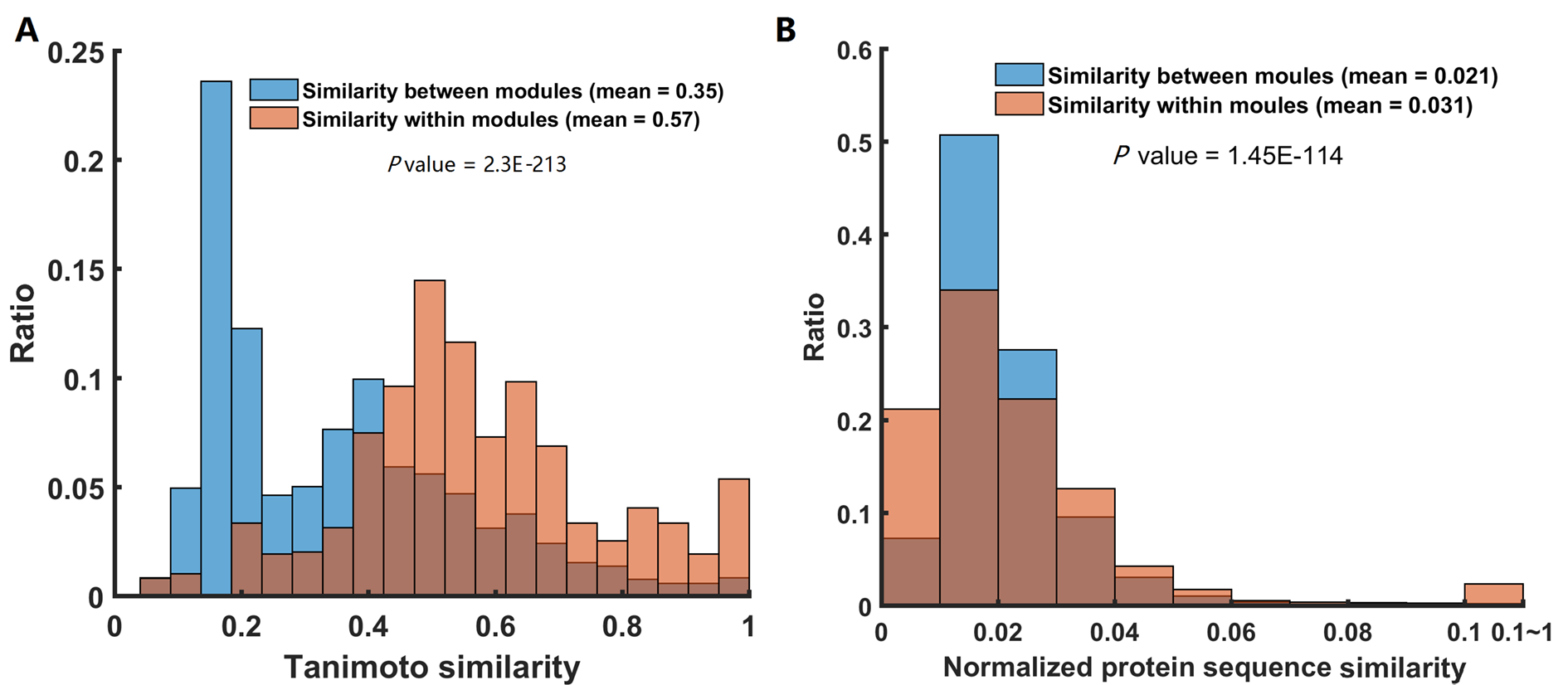

Supplement: S4 Fig — (A) The frequency histogram of molecule similarity between modules (blue) and within modules (brown). Firstly, the similarity among molecules in the same module are calculated by applying the Tanimoto similarity with their CDK fingerprints. Then, using the same method, we evaluate the molecular similarity among modules by comparing the molecules in different datasets. The result shows that mean similarity of molecules in the same module (0.57) higher than that between modules (0.35) (one-tailed student's t-test P-value = 2.3E-213). (B) The frequency histogram of target sequence similarity between modules (blue) and within modules (brown). The sequence similarity between two targets are calculated based on the Smith–Waterman sequence alignment score. The similarity score is normalized by dividing it by the geometric mean of the scores obtained from the S-score of each protein against itself. The result shows that the mean sequence similarity of proteins in the same module (0.031) higher than that between modules (0.021) (one-tailed student's t-test P-value = 1.45E-114). (TIF) [file pone.0184880.s004.tif]

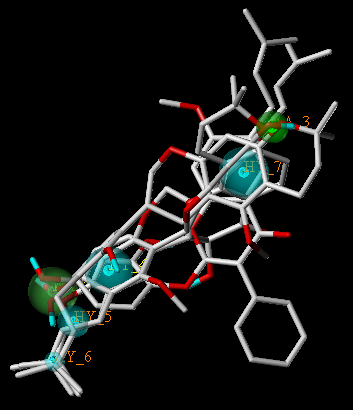

Supplement: S5 Fig — (TIF) [file pone.0184880.s005.tif]
