# Supplementary material for: Large-scale cross-species chemogenomic platform proposes a new drug discovery strategy of veterinary drug from herbal medicines
Source: PLoS One. 2017 Sep 15;12(9):e0184880. doi: 10.1371/journal.pone.0184880 (PMC5600375; doi:10.1371/journal.pone.0184880)
Supplement: S10 Table — (DOCX) [file pone.0184880.s015.docx]

Table S10. The value of SPECIFICITY, N_HITS, FEATS and PARETO for the 20 pharmacophore model of protein TBC1D1

| MODEL No. | SPECIFICITY | N_HITS | FEATS | PARETO | MODEL No. | SPECIFICITY | N_HITS | FEATS | PARETO |
| --- | --- | --- | --- | --- | --- | --- | --- | --- | --- |
| 01 | 3.982 | 4 | 7 | 0 | 11 | 3.982 | 3 | 7 | 10 |
| 02 | 3.982 | 3 | 7 | 1 | 12 | 3.982 | 3 | 7 | 11 |
| 03 | 3.982 | 3 | 7 | 2 | 13 | 3.982 | 3 | 7 | 12 |
| 04 | 3.982 | 3 | 7 | 3 | 14 | 3.982 | 3 | 7 | 13 |
| 05 | 3.982 | 3 | 7 | 4 | 15 | 3.982 | 3 | 7 | 14 |
| 06 | 3.982 | 3 | 7 | 5 | 16 | 3.982 | 3 | 7 | 15 |
| 07 | 3.982 | 3 | 7 | 6 | 17 | 3.982 | 3 | 7 | 16 |
| 08 | 3.982 | 3 | 7 | 7 | 18 | 3.982 | 3 | 7 | 17 |
| 09 | 3.982 | 3 | 7 | 8 | 19 | 3.982 | 3 | 7 | 18 |
| 10 | 3.982 | 3 | 7 | 9 | 20 | 3.982 | 3 | 7 | 19 |

* A pharmacophore model is developed for the molecules that target TBC1D1 protein in Module 1 to detailed explanation the molecular similarity in the same module. The model is generated by the GALAHAD module of the Sybyl package. And population size, mols required to hit, max generations, template molecule and alignment molecules to template individually these control parameters are respectively set to 55, 3, 50, no template and off. SPECIFICITY is a logarithmic indicator of the expected discrimination for each model. N_HITS column shows the number of ligands the pharmacophore ‘hit’ in the molecule sets. The value in the FEATS column indicates the total number of features possessed by each model. The values in the PARETO column indicate whether a model is superior to other models based on the four criteria of energy, sterics, H-bond, and MOL QRY.
